# Investigations into the Semen of Old Men

**Published:** 1869-02-01

**Authors:** A. Dieu

**Affiliations:** Physician a L’Hotel des Invalides


### *Investigations into the Semen of Old Men.*

BY A. DIEU, PHYSICIAN A L'HOTEL DES INVALIDES.

Observations have been made upon one hundred and five old men, aged from sixty-five to ninety-seven years. Beyond the age of eighty-six no spermatozoa have been found; but he accepts the case of Casper, who discovered them in a subject of ninety-six years. In sixty-four cases there were no spermatozoa in the seminal vesicles.

Sangnineous globules are frequently detected in the contents of the vesicles, traces of recent hæmorrhages, or masses of coloring matters which are the results of old hæmorrhages.

The greater part of the maladies of the reproductive organs, and of the secretory apparatus of the semen, do not appear to have any influence npon the generation of spermatozoa; however, they were absent in cases of voluminous hydrocele of the cord, in cases of voluminous varicocele, or further in indurations of the epididymis.

In a medico-legal aspect, the author considers that the existence of spermatozoa, and consequently the possibility of fecundating, may be met with in subjects no longer capable of erections.

